# COVID-19 vaccine hesitancy in five Western Balkan countries

**DOI:** 10.1093/eurpub/ckac129.736

**Published:** 2022-10-25

**Authors:** S Matovic Miljanovic, S Cvjetkovic, V Jeremic-Stojkovic, S Mandic-Rajcevic

**Affiliations:** 1 Public Health, Euro Health Group, Copenhagen, Denmark; 2 Department of Humanities, Faculty of Medicine, University of Belgrade, Belgrade, Serbia; 3 Institute of Social Medicine, Faculty of Medicine, University of Belgrade, Belgrade, Serbia

## Abstract

**Background:**

The vaccine hesitancy is a matter of global concern with inadequate global uptake postponing the moment of reaching herd immunity and bringing the COVID-19 pandemics under control. Countries in the Western Balkans struggle with vaccine hesitancy, trying to bring vaccine acceptance and ways to improve it into the focus.

**Methods:**

A cross-sectional study on vaccine hesitancy was conducted from July to September 2021 and included adult population from Albania, Bosnia and Herzegovina, North Macedonia, Montenegro and Serbia (1605 individuals). Convenience sampling was applied using anonymized online questionnaire (shared through social media) measuring, among others, trust in societal factors, social responsibility and, the credibility of information sources about COVID-19 vaccines.

**Results:**

The highest degree of trust in societal factors was found in North Macedonia (M = 3.65, SD = 1.06), followed by Montenegro (M = 3.50, SD = 1.19) and Serbia (M = 3.24, SD = 1.26). In Albania 44.7% respondents believed in reluctance of pharmaceutical companies to publish detailed research reports on the risks of adverse reactions to COVID-19 vaccines. The view that the health authorities when they encourage vaccination do so with the best intentions supported 66,3% respondents in North Macedonia and 49% in Albania and Serbia. The highest level of social responsibility (M = 4.12, SD = 1.09) was revealed in North Macedonia. Primary care physicians, health professionals in media, webpages of public health institutions, and scientific literature are the most trusted sources of information about COVID-19 in all countries.

**Conclusions:**

The study demonstrated moderate trust in societal factors and moderately high level of social responsibility in all countries. The health professionals enjoy the greatest trust, which implies that medical doctors, especially physicians in primary health care should have a pivotal role in promoting vaccination and educating the general public in the Western Balkans.

